# Spinophilin expression determines cellular growth, cancer stemness and 5-flourouracil resistance in colorectal cancer

**DOI:** 10.18632/oncotarget.2329

**Published:** 2014-08-08

**Authors:** Anna Lena Ress, Verena Stiegelbauer, Daniela Schwarzenbacher, Alexander Deutsch, Samantha Perakis, Hui Ling, Cristina Ivan, George Adrian Calin, Beate Rinner, Armin Gerger, Martin Pichler

**Affiliations:** ^1^ Division of Oncology, Department of Internal Medicine, Medical University of Graz, Austria; ^2^ Division of Haematology, Department of Internal Medicine, Medical University of Graz, Austria; ^3^ Department of Experimental Therapeutics, The University of Texas MD Anderson Cancer Center, TX, USA; ^4^ Center for RNA Interference and Non-Coding RNAs, The University of Texas MD Anderson Cancer Center, TX, USA; ^5^ Center for Medical Research, Medical University of Graz, Graz, Austria

**Keywords:** colorectal cancer, prognosis, cellular growth

## Abstract

The putative tumor suppressor gene spinophilin has been involved in cancer progression in several types of cancer. In this study, we explored the prognostic value of spinophilin expression in 162 colon adenocarcinoma patients. In addition, we generated stably expressing spinophilin-directed shRNA CRC cell lines and studied the influence of spinophilin expression on cellular phenotypes and molecular interactions. We independently confirmed that low spinophilin expression levels are associated with poor prognosis in CRC patients (*p* = 0.038). A reduction of spinophilin levels in *p53* wild-type HCT116 and *p53*-mutated Caco-2 cells led to increased cellular growth rates and anchorage-independent growth (*p*<0.05). At molecular level, reduced spinophilin levels increased the expression of the transcription factor E2F-1. In addition, we observed an increased formation of tumor spheres, increased number of CD133 positive cells and an increased resistance to 5-flourouracil (*p*<0.05). Finally, treatment with the de-methylating agent 5-aza-dC increased spinophilin expression in CRC cells (*p*<0.05), corroborated by a correlation of spinophilin expression and extent of methylated CpG sites in the gene promoter region (p<0.001). In conclusion, gain of aggressive biological properties of CRC cells including cellular growth, cancer stem cell features and 5-flourouracil resistance partly explains the role of spinophilin in CRC.

## INTRODUCTION

Despite a decrease of incidence and mortality in the last years, colorectal cancer (CRC) still remains the most common cancer of the digestive system, associated with high morbidity and mortality rates according to the American Cancer Society in 2014 [1]. Depending on the clinical stage at diagnosis, different treatment approaches including surgery, radiotherapy and chemotherapy are the mainstay of the therapeutic armamentarium [2]. The introduction of novel targeted agents in CRC treatment within the last 10 years improved the survival, though metastatic CRC represents an incurable disease in most cases. Hence, there is a real need for the discovery of novel prognostic and pathogenesis-driving factors to improve clinical outcome of these patients [3-5]. Spinophilin (also known as PPP1R9B and neurabin II) is a multifunctional scaffold protein located at chromosome 17q21.33. This chromosomal region is frequently associated with microsatellite instability and loss of heterozygosity (LOH), features frequently associated in the pathogenesis of CRC [6, 7]. Previous studies also reported that low levels of spinophilin expression in tumors are associated with cancer progression and poor prognosis in lung, kidney, head and neck cancer, and leukemia [6, 8]. Ferrer and colleagues demonstrated in mouse embryonic fibroblasts that the loss of spinophilin contributes to high levels of phosphorylated retinoblastoma protein (pRb) with the consequence of increased activation of the tumor suppressor *p53*. In the absence of *p53* or impaired function by mutation, the cells develop a pro-proliferative phenotype, given that the elevated pRb, which is normally neutralized by *p53*, drives a pro-proliferative state [9]. However, in two recently published studies of hepatocellular carcinoma and CRC, loss of spinophilin was associated with *p53-*independent cellular proliferation [10, 11]. In the first previously published study of spinophilin in CRC, Estevez-Garcia *et al.* recently described a correlation of spinophilin expression and advanced tumor stages. Furthermore, low spinophilin expression was associated with a more aggressive histologic phenotype, faster relapse and poorer survival in their study [11]. However, an external validation of these findings is still missing and the possible underlying cellular and molecular mechanisms in CRC are poorly explored. Importantly, the external validation of such newly identified prognostic risk factors in independent cohorts of patients is paramount prior to the generalization of the applicability of a prognostic marker and some studies fail to replicate the findings of others [12, 13]. Therefore, we validated the influence of spinophilin expression on survival in CRC on a large independent cohort of patients. Based on the positive clinical data, we further studied the potential cellular functions and molecular mechanisms of alterations in spinophilin expression in CRC.

## RESULTS

**Figure 1:**
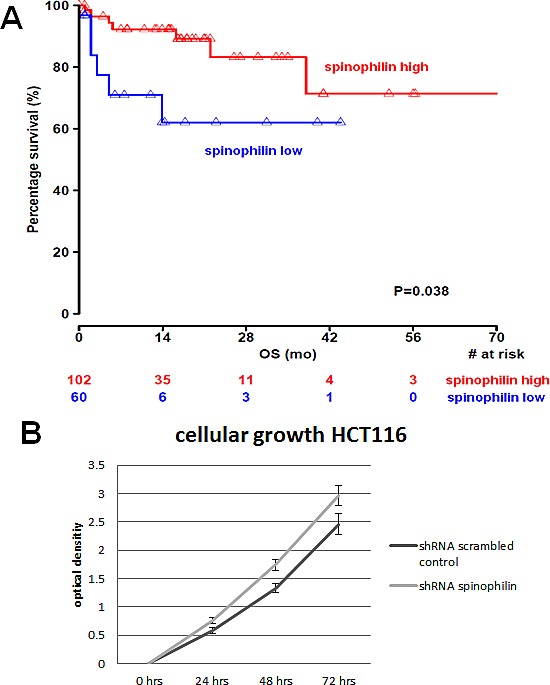
Spinophilin expression and influence on survival and cellular growth **(A)** In 162 available patients of The Cancer Genome Atlas data set, a low spinophilin expression is associated with poor survival. **(B)** Silencing of spinophilin by shRNA in HCT116 cells leads to increased cellular growth rate in the WST-1 assay (0 to 72 hours, *p*<0.05).

**Figure 2:**
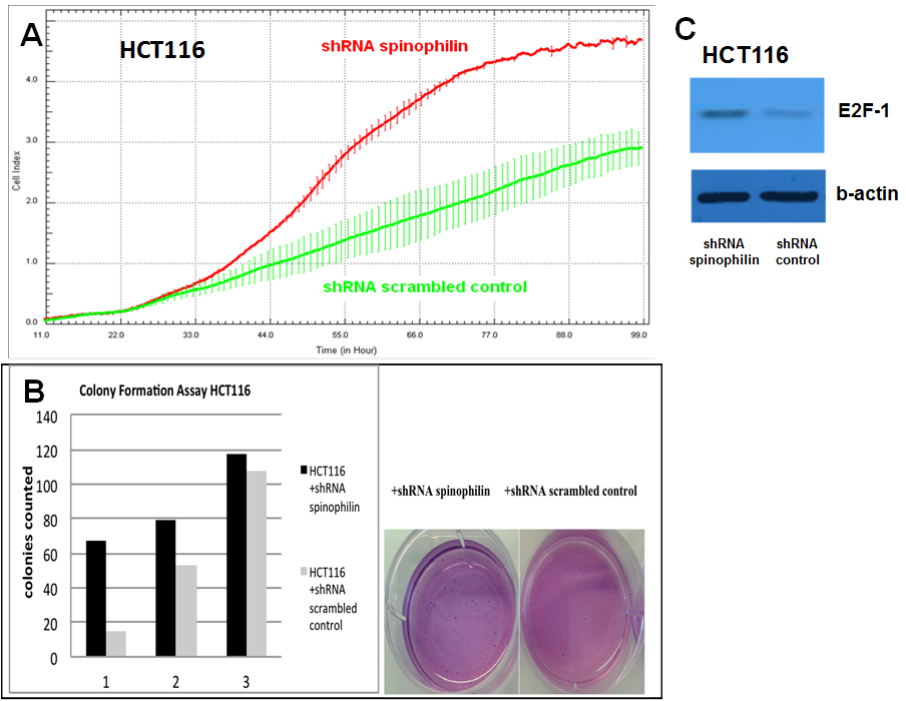
Cellular growth, anchorage-independent growth and molecular alterations **(A)** In the xCELLigence system, increase in the cellular growth is observed in HCT116 shRNA spinophilin (red, upper line) compared to HCT116 shRNA scrambled control (green, lower line) cells **(B)** Colony numbers in soft agar plates are significantly increased in the spinophilin-silenced HCT116 cells. Numbers below the bars are representing three independent experiments. **(C)** In the spinophilin-silenced cells increased amount of E2F-1 was detected in Western blot analysis.

**Figure 3:**
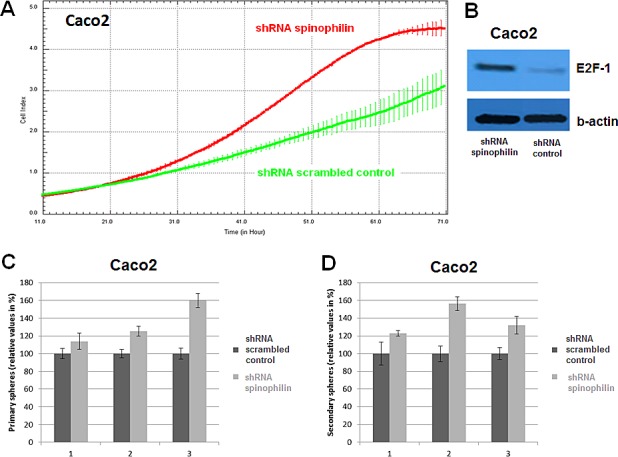
Cellular growth and tumor sphere formation **(A)** A significant increase in cellular growth of spinophilin-silenced cells (green line) could also be detected in the Caco-2 cell line and **(B)** increased E2F-1 protein expression. **(C-D)** A significant increase in primary and secondary tumor spheres under low attachment conditions, which is indicative for increased self-renewal capacity, could be observed in the spinophilin-silenced cells.

**Figure 4:**
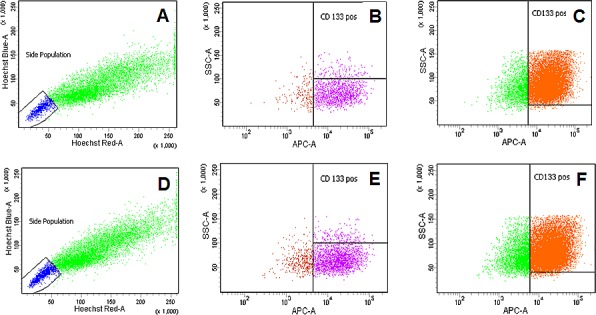
Representative scatter dot-plot images of flow cytometry for CD133 positive total population, side population and CD133 positive side population **(A-C)** Caco2 shRNA scrambled control cells: (A) Hoechst side population staining: 1.6 % of total population (B) CD133-APC staining gated on side population: 1.4% of total population (C) CD133 gated on total population: 11.8% total population **(D-F)** Caco2 shRNA spinophilin cells: (D) Hoechst side population staining: 4.1 % of total population (E) CD133-APC staining gated on side population: 3.7% of total population (F) CD133 gated on total population: 21.8% total population.

**Figure 5:**
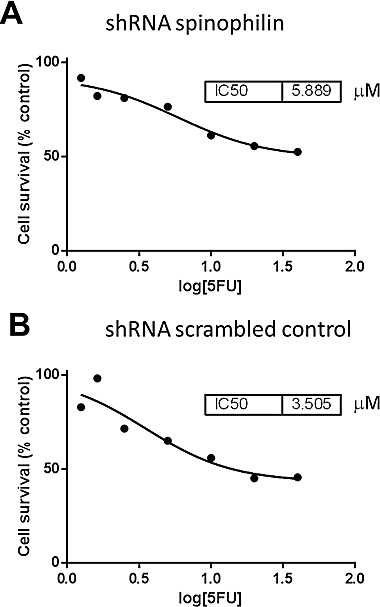
Dose-response curves and IC50 values for 5-flourouracil treated Caco2 cells **(A)** The shRNA spinophilin silenced cells show a higher IC50 value compared to **(B)** the shRNA scrambled control cells.

First of all, we measured spinophilin expression in eleven different CRC cell lines. Overall, in seven out of eleven CRC cell lines spinophilin was expressed at a lower level compared to normal colon (Supplementary Figure S1). This lower expression in CRC cell lines prompted us to expand our analysis on *in vivo* data of CRC patients. For evaluation of the prognostic relevance of spinophilin expression in an external data set, we analyzed mRNA expression data from the TCGA dataset. In 162 available colon adenocarcinoma patients (Supplementary Table S1), we found that a lower expression of spinophilin is associated with poor patient survival (*p* = 0.038, Kaplan Meier curve Figure 1A). In our study cohort, low spinophilin expression levels did not significantly correlate with patient age, gender, location, clinical stage, primary tumor stage, distant metastases or microsatellite status (all *p*-values >0.05, Supplementary Table S2). Univariate analysis identified advanced clinical stage (stage I+II versus III+IV) and low expression spinophilin expression as poor prognostic factors for overall survival (all *p*-values <0.05), whereas age, gender, location of the tumor and microsatellite status were not significantly associated with survival (data not shown). To test whether the prognostic value of low spinophilin expression is independent of other risk factors of poor survival, a multivariate analysis was performed using a Cox proportional hazard model. Multivariate analysis including all factors significant in univariate analysis (clinical stage and level of spinophilin expression) confirmed low spinophilin expression as an independent predictor for poor survival in CRC patients (hazard ratio = 3.02, 95% confidence interval =1.04-8.79, *p*<0.042). For a more detailed characterization of the possible biological impact of low spinophilin levels in CRC cells, we generated stably spinophilin-silenced CRC cell lines. For this purpose, we selected the spinophilin high expressing HCT116 and the spinophilin low expressing Caco-2 cell line (Supplementary Figure S1). These two cell lines are also different with regard to their *p53* mutational status (HCT116: *p53* wild-type and Caco-2: *p53* mutated, respectively). Generating stably expressing spinophilin-directed shRNA cells, a relative reduction to about 60% expression levels could be detected (Supplementary Figure S2). Consecutively, we explored the growth behavior of the spinophilin-silenced cells using the WST-1 proliferation assay. In the *p53* wild-type HCT-116 cell line we observed a statistically significant increase of cellular growth in spinophilin-silenced cells over a 72 hours observational period (Figure 1B). To substantiate these findings with a second independent method, we monitored cellular growth rates by using the xCELLigence system. This real-time growth assay continuously detects the well impedance as a measure of cell density. As shown in Figure 2A cellular growth (indicated by the cell index on the Y-axis) increases in cells with reduced spinophilin expression compared to the control cells. We further characterized the influence of spinophilin expression levels on well-established features of cancer stem cells. Anchorage-independent growth was assessed by soft agar colony formation assay. A significantly higher number of colonies in spinophilin-silenced cells compared to control cells in HCT-116 cells could be observed (*p* = 0.012, Wilcoxon test, Figure 2B). To investigate whether differences of apoptotic activity are responsible for the differently observed growth rates and colony formation, we performed an annexin staining using a flow cytometer. The shRNA scrambled-control cell line showed no increased proportion of this apoptosis-related marker (Supplementary Figure S3A, B). In addition, we measured the full length and cleaved PARP protein expression by Western Blot (Supplementary Figure S3C), the gene expression of the pro-apoptotic *Bax* gene and the anti-apoptotic *Bcl-2* gene, and the EMT-related markers E-cadherin and vimentin, but could not detect any significant differences (data not shown). To explore possible molecular down-stream effects of spinophilin-silencing, we observed higher levels of E2F-1, a well-known driving factor for cellular growth (Figure 2C). Using a second cell line, the *p53*-mutated Caco-2 cell line, we independently confirmed that lower spinophilin expression levels are associated with increased cellular growth rates and increased E2F-1 protein expression (Figure 3A and 3B). For phosphorylated retinoblastoma protein we received rather inconsistent findings with regard to activation of this protein (data not shown). To further clarify whether *p53* mutation status influence the behavior of CRC with varying levels of spinophilin expression, we added a survival analysis in the 162 TCGA dataset patients by stratifying them into four groups according to *p53*-mutational status and spinophilin expression (details see also in the Material and Methods section). However, we could not detect any influence of *p53* mutations when added to the spinophilin expression levels on a better separation of survival curves (data not shown). Next, we investigated the effect of spinophilin-silencing on self-renewal capacity using tumor sphere assays and analyzed the expression of the CD133 surface marker and proportion of the side population by FACS. As the HCT116 cell line did not generate tumor spheres in sufficient numbers to count and did not show any side population in FACS analysis, we performed this step with Caco-2 cells only. The number and size of primary (Figure 3C) and secondary (Figure 3D) tumor spheres were significantly higher in spinophilin-silenced cells (*p* <0.05). We also observed a numerically increase of CD133 positive cells in the whole cell population (14.97% versus 8.23%) as well as in the sorted side population for spinophilin-silenced cells (3.27% versus 2.17%, *p*<0.05). Detailed scatterplot images of the different subpopulations are shown in Figure 4A-F. Consequently, exposing these cells to different concentrations of 5-FU for 48 hours, we observed a higher resistance to 5-FU in the spinophilin-silenced cells. Figure 5 shows the dose-response curves and corresponding IC50 values. In order to evaluate some molecular causes for the different spinophilin expression levels in tumor tissue, we analyzed the TCGA dataset with regard to copy number changes in the spinophilin gene region. For this purpose, we downloaded copy number information from the cBioportal for cancer genomics as well as copy number data as generated by the GISTIC algorithm. This algorithm attempts to identify significantly altered regions of amplification or deletion across sets of patients. It generates putative gene/patient copy number specific calls: “-2” is a deep loss, possibly a homozygous deletion, “-1” is a single-copy loss (heterozygous deletion), “0” is diploid, “1” indicates a low-level gain, and “2” is a high-level amplification. Of the 155 patients with all available data in our cohort (copy number changes and expression values), 22 (14.2%) had “-1” (i.e. single-copy loss) and 28 (18.1%) had “+1” (i.e. low-level gain). Interestingly, we could see a direct correlation with regard to spinophilin expression/copy numbers in tumor samples (R = 0.38, *p*<0.001, Supplementary Figure S4).

**Figure 6:**
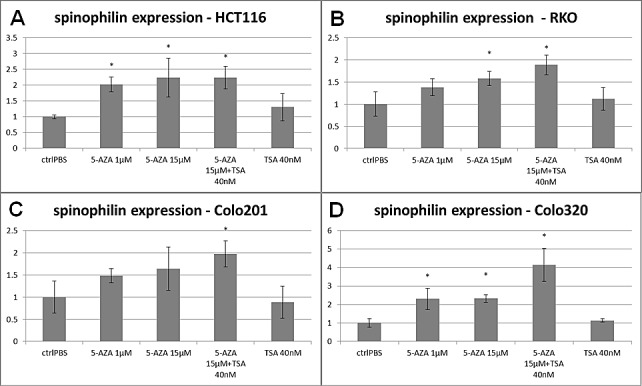
Spinophilin expression after de-methylation with 5-aza-dC Four different cell lines, (A) HCT-116, (B) RKO, (C) Colo320 and (D) Colo201 cells showed an increase in spinophilin expression after 96 hours of treatment with 5-aza-dC or even enhanced by the histon-deacetylase inhibitor trichostatin A. Treatment with the histon-deacetylase inhibitor trichostatin A alone had no effect on spinophilin expression.

Finally, in order to determine whether epigenetic changes influence spinophilin expression in CRC, we treated the HCT116, RKO, Colo320 and Colo201 cell lines with the de-methylation agent 5-aza-dC and the histone deacetylase inhibitor trichostatin A(TSA). After treatment with these agents a significant up-regulation of spinophilin in these cell lines at a varying extent (up to 4 fold) was observed after 15μM 5-aza-dC or combined TSA treatment but not after TSA only treatment (Figure 6). To explore the role of different levels of CpG methylation in the promoter region of spinophilin *in vivo*, we utilized bioinformatic tools to retrieve data for 19 CpGs sites in a CpG island in the promoter region of the spinophilin gene (Position: chr17:48226224-48228625, Band: 17q21.33, Genomic Size: 2402 bp, Supplementary Figure S5A). We used data generated by an Illumina Infinium Methylation450 platform derived from the TCGA data set. We obtained methylation data of 31 patients out of the 162 in our cohort. In these patients, we found for at least two CpGs in this region a significantly negative correlation between spinophilin expression and DNA methylation extent (Supplementary Figure S5B-C).

## DISCUSSION

Tremendous efforts have been spent on the exploration of the underlying molecular mechanisms in tumor progression and metastases of CRC. This initiative has led to the discovery of some key molecules and pathways including the vascular endothelial growth factor (VEGF) in tumor angiogenesis and the epidermal growth factor receptor (EGFR) with the therapy guiding *KRAS/NRAS* mutations. These and other molecules highly impacted the clinical practice of CRC treatment [14, 15]. Nevertheless, due to the moderate survival rates of metastatic CRC, the race for identifying novel factors, whether useful as drug targets, prognostic markers or diagnostics, is still ongoing [16-19]. In this study, we report about the role of spinophilin, a previously recognized novel tumor suppressor gene, in CRC. First, we found using the internationally established TCGA dataset that low spinophilin levels are associated with poor survival in uni- as well as multivariate analyses. These findings match well with the previous report about spinophilin in CRC, where Estevez-Garcia and colleagues proposed a negative influence on survival and relapse-free time especially in stage III CRC patients treated with adjuvant fluoropyrimidine therapy [11]. In this well-designed study, the authors also report about lower response rates to therapy in patients with low spinophilin expression. In addition, using CRC cell lines (Colo205, HT29, SW480) with different *p53* mutational status (wild-type or mutated), which were different from the ones we used in our study, they also observed an increased growth in spinophilin-silenced cells in soft agar assays. A clinical or experimental observation holds true and gets substantiated when independent models and groups confirm the results [12, 13]. Therefore, the findings in our study confirm that low spinophilin expression is associated with poor survival and, by using different cellular growth assays and cell lines, a loss of this protein drives cellular growth in CRC independent of the p53 mutational status. In our study, we further demonstrated that reduction of spinophilin leads to an increase of the transcription factor E2F-1. This mechanism of action seems to be similar as previously reported by Ferrer *et al.* [9]. The higher proliferative state of tumors with low spinophilin expression might be one explanation for the poor clinical outcome in this group of patients. However, in a further attempt to characterize the cellular mode of action, we also identified that spinophilin-silenced cells are prone to form more tumor spheres under ultra-low attachment conditions than control cells. Furthermore, a higher number of CD133 positive cells in the whole population as well as in the side population could be identified. Both of these features, tumor sphere formation and CD133 positive cells/side population have been previously described as cancer stem cell features in CRC [20]. In our study, we also found a significant correlation between copy number changes and the levels of spinophilin expression. Estevez-Garcia and colleagues hypothesized in their study that spinophilin might increase the resistance to chemotherapeutic agents [11]. In line with this notion, we detected higher growth rates of 5-FU treated cells in the spinophilin-silenced cells, which support this hypothesis. Interestingly, we also observed an up-regulation of spinophilin in CRC cells after treatment of the DNA-demethylation agent 5-aza-dC. Estevez-Garcia *et al*. detected no DNA methylation in the promoter region of spinophilin gene. However, they used methylation-specific PCR, which only covers a limited number of CpG sites and is a rather qualitative method for DNA-methylation detection. In our study, we used genome-wide methylation data and found for at least two CpG sites a negative correlation between extent of methylation and spinophilin expression. According to our data, either the region of the promoter or some other enhancer regions of the spinophilin gene are affected by DNA methylation, which in turn can influence the expression level of this tumor suppressor in tumors. Another explanation for our observation might be an indirect effect of other trans-acting transcription factors that are activated by the DNA-demethylating agent and thus increase the transcription of the spinophilin locus. Clinically, this relationship of low spinophilin levels and aggressive behavior may be circumvented by restoration of spinophilin expression or, even easier to perform, the inhibition of down-stream affected factors like E2F-1 [21]. However, further research to decipher additional modes of action and pre-clinical trials are necessary before the true value of spinophilin-targeting in clinical practice can be estimated.

In summary, we confirmed the previously identified association of low spinophilin expression and poor prognosis in CRC patients. In addition, we found that spinophilin might be involved in cancer stem cells, that the E2F-1 signaling is positively influenced and that DNA methylation in CRC cells might explain in part the differential expression of spinophilin in CRC.

## MATERIALS AND METHODS

### Patient cohort

For validation of spinophilin expression and its prognostic relevance in CRC tissue, we made use and analyzed data publicly available from the Cancer Genome Atlas Project (TCGA; http://tcga-data.nci.nih.gov/) for CRC patients [22]. For 162 patients with colon adenocarcinoma (COAD), we acquired from TCGA portal mRNA expression (level 3 RNASeq v2 GA) and clinical information. The clinical information was completed from the previously published study [22]. For 151 patients among them, we obtained *p53* somatic mutation information from same study [22]. Analyses were carried out in R statistical environment (version 3.0.1) (http:///www.r-project.org/). All tests were two-sided and considered statistical significant at the 0.05 level. For prognostic studies, we checked for a relationship between spinophilin mRNA expression and overall survival as follows: Patients were grouped into percentiles according to mRNA expression. The log-rank test was employed to determine the association between mRNA expression and overall survival. The Kaplan-Meier method was used to generate overall survival curves. The cut-off to optimally separate the patients into low/high groups (log-rank test p-value minimum) was chosen. The cut-off value to optimally separate the patients in this cohort was determined at 0.34 (where 1 means 100% of the cohort). Based on the previous description that a loss of spinophilin led to tumor progression especially in a *p53*-mutated genotype [9], we considered whether adding *p53* mutation status added information to the prognostic value. For this purpose, we did this by letting the spinophilin cut-off level to vary between 0.25 and 0.75 and recording for each patient *p53* mutation status. This splits the cohort in four groups corresponding to low/high spinophilin expression and mutated/wild-type *p53* status. The log-rank test was again employed to determine the association between spinophilin mRNA expression/*p53* status and overall survival.

For testing the CpG methylation status of CpGs in a CpG island in the promoter region of the spinophilin gene (position: chr17:48226224-48228625, Band: 17q21.33, Genomic Size: 2402 bp) we used data generated by an Illumina platform (Infinium Methylation450 platform) derived from the TCGA data set. We obtained methylation data of 31 patients out of the 162 in our cohort for 19 CpG sites. The reason for the limited number of patients was that methylation profiling in the TCGA dataset was performed for some patients on an Infinium Methylation27 platform and for some patients ( n = 31) on an Infinium Methylation450 platform. Unfortunately, the Infinium Methylation27 package does not have a comprehensive coverage as the Infinium Methylation450 and the spinophilin promoter region was not targeted on the Infinium Methylation 27 platform. For quantifying the methylation levels, the analysis resulted in beta-values. A beta-value is generated by Illumina′s technology. Beta values for each interrogated CpG locus are calculated as Beta = M/(M+U)+100, whereby M and U represent the mean signal intensities for replicate methylated (M) and unmethylated (U) probes on the array. Beta can take a value between 0 (no methylation) and 1 (highest degree of methylation).

### Cell culture

The human CRC cell lines Caco-2 and HCT-116, which are extensively used in this study, were purchased from American Type Culture Collection (Manassas, CA, USA) and their origin was proven by DNA identity STR-analysis. Caco-2 cells were grown in MEM with Earle's Salts and L-Glutamine. For HCT-116 McCoy's 5A modified Medium (w/o L-Glutamine, 2,2g/L sodium bicarbonate) was used. Growth medium contained 10% foetal bovine serum gold (PAA Laboratories, Pasching, Austria) and antibiotics (penicillin and streptomycin). Cells were incubated in a 5% CO_2_ humidified atmosphere at 37°C. All other CRC cell lines (SW480, RKO, DLD-1, Colo205, Colo201, LS174, Colo320, SW620, HT29) were obtained from the American Type Culture Collection or other commercially suppliers and were grown under the recommended standard conditions. For comparison of spinophilin mRNA expression in these cell lines to normal human colon, we used Human Colon Total RNA (Clontech, catalog no. 636553).

### ShRNA lentiviral particles transduction

Caco-2 and HCT-116 cells were seeded in 6-well plates 24 hours prior to viral infection and incubated overnight in 2 ml of complete growth medium containing 10% FBS and 1% antibiotics. On the day of transfection the medium was removed and 2 ml of complete growth medium containing 8 μg/ml polybrene (Santa Cruz Biotechnology, Santa Cruz, CA) and 10 μl of ViralPlus Transduction Enhancer (ABM, Richmond, BC, Canada) were added. Cells were infected by adding 50 μl of shRNA spinophilin (Sense strand: 5’-GGGAGGUGCGCAAGAUUAATT-3’, Antisense strand: 5’-UUAAUCUUGCGCACCUCCCGG-3’) lentiviral particles (ABM) or shRNA scrambled control lentiviral particles (ABM), respectively. Stably transfected Caco-2 cells were selected with 7.5 μg/ml puromycin dihydrochloride (Gibco, Carlsbad, CA) and HCT-116 cells were selected by adding 0.5 μg/ml puromycin dihydrochloride.

### Quantitative RT-PCR analysis of spinophilin expression level

For detection of spinophilin expression from all cell lines described in this study, RNA was extracted by using a Trizol standard extraction protocol and 1 μg of total RNA was reverse transcribed by the QuantiTect Reverse Trascription Kit (Qiagen, Hilden, Germany) according to the manufacture′s protocol. Quantitative RT-PCR was carried out in triplicates for each sample using commercially available primers specific for spinophilin (Hs_PPP1R9B_1_SG QuantiTect primer assay, Qiagen), EMT-related genes (E-cadherin, Hs_CDH1_1_SG QuantiTect primer assay, Qiagen; Vimentin, Hs_VIM_1_SG QuantiTect primer assay, Qiagen) and specific primers for the housekeeping genes GAPDH (forward primer: AAG GTC GGA GTC AAC GGA TTT; reverse primer: ACC AGA GTT AAA AGC AGC CCT G) and B2M (forward primer: TGC TGT CTC CAT GTT TGA TGT ATC T; reverse primer: TCT CTG CTC CCC ACC TCT AAG T) on a LightCycler® 480 Real-Time PCR System (Roche Diagnostics, Mannheim, Germany) using QuantiTect SYBR Green PCR Kit (Qiagen). The geometric mean of the housekeeping genes GAPDH and B2M was used for normalization and relative gene expression levels were calculated according to the 2^-ΔΔCT^ method.

### Western blot

Total proteins from stably transfected HCT-116 and Caco-2 cells were extracted with radioimmunoprecipitation assay (RIPA) buffer (150 mM NaCl, 50 mM Tris-HCl, pH 7.5, 1% Triton, 0.1% SDS, 0.1% sodium deoxycholate and 1% Nonidet P40). 25 μg of total cellular proteins were resuspended in laemmli buffer (4% SDS, 20% glycerol, 10% 2-mercaptoethanol, 0.004% bromphenol blue and 0.125 M Tris HCl, pH approx. 6.8) and heated at 95 °C for 5 minutes. Proteins were separated by a 4–15% Mini-PROTEAN® TGX™Precast Gel (Biorad, Hercules, CA) and transferred onto a nitrocellulose membrane (Applichem, St. Louis, MO). The membrane was blocked for one hour with 3% non-fat dry milk in Tris buffered Saline/0.1% Tween-20. Immunoblotting was performed and antibodies specific for spinophilin (Cell Signaling, Danvers, MA, diluted 1:1000 in 1% non-fat dry milk in Tris buffered Saline/0.1% Tween-20 ), pRb (directed against phosphorylated serine 807/8, Cell Signaling, diluted 1:1000 in 1% non-fat dry milk in Tris buffered Saline/0.1% Tween-20), E2F-1 (Cell Signaling, diluted 1:1000 in 1% non-fat dry milk in Tris buffered Saline/0.1% Tween-20) and β-Actin (Sigma, diluted 1:5000 in 1% non-fat dry milk in Tris buffered Saline/0.1% Tween-20 ) were detected using HRP-conjugated anti-mouse or anti-rabbit antibodies, respectively (Dako, Glostrup, Denmark). Visualization was performed using an enhanced chemoluminescence detection system (Super Signal West Pico, Thermo Scientific, Rockford, IL).

### WST-1 proliferation assay and 5-flourouracil resistance assay

To test whether altered spinophilin expression influences cellular growth rates of CRC cells, we applied the WST-1 proliferation assay (Roche Applied Science, Mannheim, Germany). In more detail, after standard trypsinization 2x10^4^ HCT-116 cells (shRNA spinophilin and shRNA scrambled control) per well were seeded in a 96-well culture plate. The cells were incubated in normal McCoy`s 5A modified Medium growth medium (Biochrom, Berlin Germany) for 48 hours and the WST-1 proliferation reagent (Roche Applied Science, Mannheim Germany) was applied according to the manufacturer's recommendations. After four hours the colorimetric changes were measured using a SpectraMax Plus (Molecular Devices, CA, US) at a wavelength of 450 nm with a reference wavelength at 620 nm. Three independent experiments in six technical replicates were performed. The same assay was used for testing the resistance of Caco2 cells to 5-fluorouracil (5-FU). In detail, 24 hours after seeding the spinophilin-silenced and control cells to wells of a 96-well plate, eight different two-fold diluted concentrations of 5-FU including one untreated control were added to the cells (in the range of physiological levels between 0.625 to 40 μM). After 48 hours, the WST-1 assay was applied and the optical density compared to the untreated cells was calculated. Dose-response curves and IC50 values were calculated by using the software tools of Prism GraphPad software version 6.0.

### xCELLigence cellular growth assay

A second independent cellular growth assay was used to substantiate the findings of the WST-1 assay. Labeling-free cell-based assay system (xCELLigence system, Roche Applied Science, Mannheim Germany) was used for real-time analysis of cellular growth of HCT-116 and Caco-2 shRNA transfected and control cells. 1x10^4^ HCT-116 cells and 2x10^4^ Caco-2 cells were seeded in electronic microtiter plates (E-Plate™; Roche Diagnostic, Mannheim, Germany) and measured for 66 h with the xCELLigence system according to the instructions in the user's manual. Cell density measurements were performed three times in triplicates with a programmed signal detection every 15 min. Data acquisition and analyses were performed with the RTCA software (version 1.2, Roche Diagnostics).

### Apoptosis, CD133 staining and side population

10^6^ cells/ml were resuspended in 1x binding buffer (BioLegend, London, UK). 100 μl cell suspension was stained with 5μl AnnexinV-APC (BioLegend) and 10 μl SYTO ® 24 Green Fluorescent Nucleic Acid Stain (Molecular Probes) incubated at room temperature for 5 min in the dark. Flow cytometry analyses of HCT-116 cells were performed with FACS LSRII (BD Biosciences, San Jose, US). 10000 events were collected and identified in the side and forward scatter with linear scale. Fluorescence signal was analyzed with logarithmic scale, analyses were done with FACS Diva software (BD), graphical analyses were performed in FCS express (De Novo Software, Los Angeles, US).

To identify the side population and non-side population fractions, cells were trypsinized and suspended at 1x10^6^ cells/ml in culture medium containing 2% FBS. Hoechst 33342 dye (Sigma-Aldrich, Vienna, Austria) was added at a final concentration of 5 μg/ml in the presence or absence of 100 μM verapamil (Sigma-Aldrich), and the cells were incubated at 37°C for 120 min with intermittent shaking every 15 min. The cells were then stained with 2.5 μl CD133-APC antibody (Milteny Biotec, Bergisch Gladbach, Germany) and incubated in the dark for 30 min on ice. The cells were washed twice with PBS and resuspended in 250 μl PBS with 2 μg/ml Propidium iodide (PI) and kept on ice until FACS analysis. Cell analyses were carried out using FACS LSRII (BD). Hoechst 33342 dye was excited by a 350-nm ultraviolet laser, and fluorescence emission was dual wavelength analyzed (Hoechst blue 440/40; Hoechst Red 695/40). In general, cells in a side population have distinguishing biological characteristics, commonly indicated by the ability to efflux chemotherapy drugs or other marker molecules (Hoechst 33342). This side population is therefore also regarded as a distinct population of cells, commonly enriched by cells with cancer stem cell features (which includes by definition a higher degree of chemoresistance).

### 5-aza-dC and trichostatin A treatment to explore epigenetic effects

To evaluate whether spinophilin is directly or indirectly regulated by DNA-hypermethylation or histon-acetylation we treated HCT-116, RKO, Colo320 and Colo201 CRC cells with modifiers of these epigenetic changes. De-methylation was induced with 5-aza-dC (Sigma-Aldrich, St. Lois, US) treatment at different concentrations, which were previously proven to induce maximal de-methylation of the DNA without killing the cells. 100000 cells per well were seeded in 6-well culture dishes and 24 hours later the drug treatment was started. Culture media for HCT-116 and RKO cells contained 1 μM or 15 μM 5-aza-dC. Cells were incubated for 96 hours with 5-aza-dC with the culture media being replaced every 24 hours with fresh media containing 5-aza-dC. RNA was extracted after 96 hours of drug treatment. The experiment was also performed with cells continuously exposed to 40 nM trichostatin A (TSA; Sigma-Aldrich) without 5-aza-dC and as an add-on combinatorial treatment after 72 hours of 15 μM 5-aza-dC to assess the effect of histone acetylation on DNA re-methylation. This chosen concentration has previously been shown to cause hyper-acetylation in HCT-116 cell line [23].

### Anchorage-independent growth

The efficiency of colony formation of stably transfected CRC cells in soft agar was determined by plating 2500 cells in 1 ml of complete growth medium containing 0.35% low gelling temperature agarose (Sigma-Aldrich, Seelze, Germany) over 1.5 ml of growth medium containing 0.5% agar (Sigma) in a 35mm dish. Cells were cultured at 37°C and 5% CO_2_ for up to four weeks. Colonies were stained with 0.005% crystal violet (Sigma) in 25% methanol and the number of colonies was counted using a dissecting microscope.

### Colon sphere formation assay

To assess colon sphere formation, we performed a spheroid growth model as previously described with slight modifications [24]. In detail, the adherent growing CRC cell lines were dissociated into single cells using trypsin/EDTA and seeded in ultra-low attachment 6-well plates (Corning, NY, USA) in serum-free medium (SFM). Caco-2 colon spheres were seeded in SFM MEM with Earle's salts containing 2 mmol/L L-glutamine (PAA), supplemented with 1xB27 supplement (Gibco), 20 ng/ml human epidermal growth factor EGF (PeproTech), 20 ng/ml human basic fibroblast growth factor FGF (PeproTech), 2 mM sodium pyruvate (PAA), 1% non-essential amino acids (PAA), 4IU/l insulin actrapid (Novo Nordisk) and 1% antibiotic/antimycotic solution (Sigma-Aldrich). Primary colon spheres were counted under a microscope ten days after seeding single cells. A sphere formation assay of secondary spheres was done by using primary spheres. Briefly, primary spheres were dissociated into single cells using TrypLE Select (Gibco, Invitrogen Life Technologies). Single-cell suspensions of colon spheres were seeded at 2.000 cells per well in SFM plus supplements. Ten days after seeding secondary spheres were counted. Three independent experiments with each three technical replicates were performed.

### Statistical analysis

All data represent mean values of at least three independent experiments ± SEM (standard error of mean). Student′s unpaired t-test or non-parametric tests were used to evaluate differences between groups. *P-*values below 0.05 were considered statistically significant.

Overall survival (OS) was defined as the time from date of diagnosis to the date of death of any cause. Cox proportional hazard models were performed using SPSS version 19.0 software (SPSS Inc., Chicago, IL, USA). Univariate Cox proportional hazards models including age, gender, tumor stage, microsatellite status, location and spinophilin expression were used to determine the clinico-pathological parameters that were statistically significant for OS. The reported results included hazard ratios (HR) and 95% confidence intervals (CI).

## SUPPLEMENTARY MATERIAL, FIGURES AND TABLES


