# Supplementary material for: Spinophilin expression determines cellular growth, cancer stemness and 5-flourouracil resistance in colorectal cancer
Source: Oncotarget. 2014 Aug 8;5(18):8492–502. doi: 10.18632/oncotarget.2329 (PMC4226699; doi:10.18632/oncotarget.2329)
Supplement: Supplementary file 1 [file oncotarget-05-8492-s001.pdf]

Spinophilin expression determines cellular growth, cancer stemness and 5-flourouracil resistance in colorectal cancer

Supplementary Material

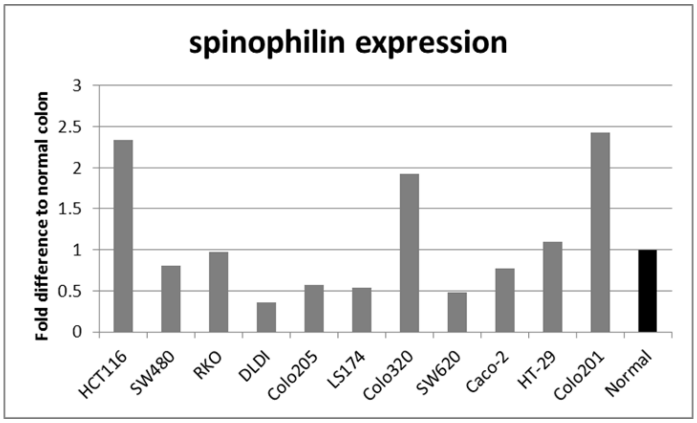

Supplementary Figure S1: Spinophilin expression in eleven different colorectal cancer cell lines. Seven out of eleven cell lines showed a lower expression in comparison to normal colon tissue.

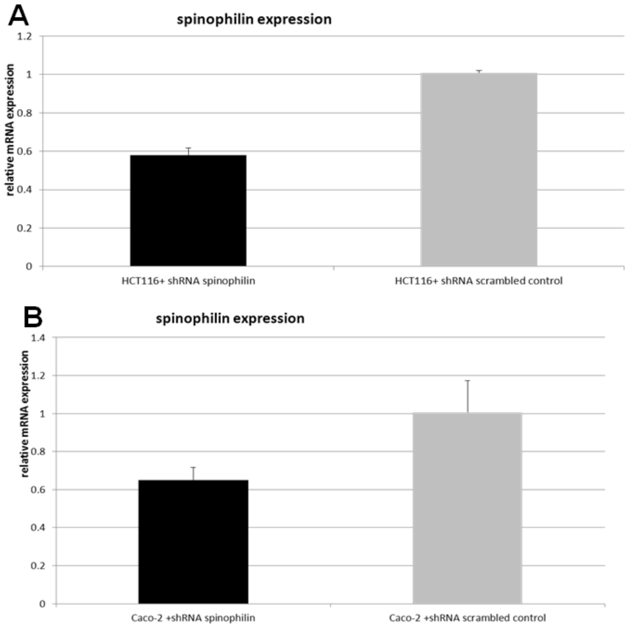

Supplementary Figure S2: Spinophilin expression after shRNA-targeted silencing. **(A)** HCT-116 and **(B)** Caco-2 cells showed about 60% of spinophilin levels compared to scrambled control treated cells.

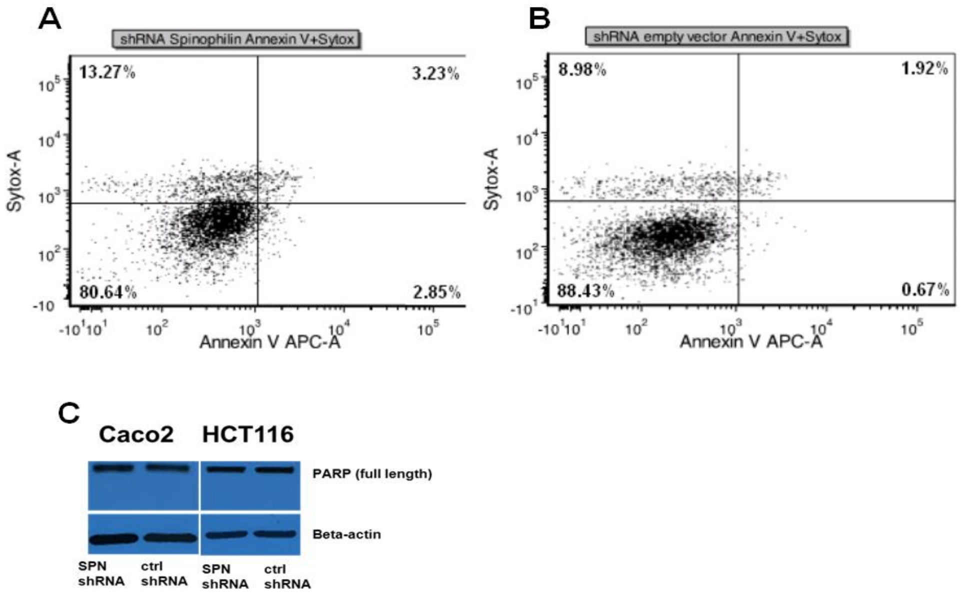

Supplementary Figure S3: Apoptotic activity in spinophilin-silenced cells. (A) HCT-116 shRNA treated cells and **(B)** control cells were analyzed with Annexin V and Sytox staining using FACS LSRII. This exhibited no significant difference in the population with AnnexinV/Sytox staining and indicates no apoptotic effects at all. **(C)** A Western blot analysis also showed no differences in PARP cleavage in both cell lines comparing spinophilin-silenced (SPN shRNA) and control cells (ctrl shRNA).

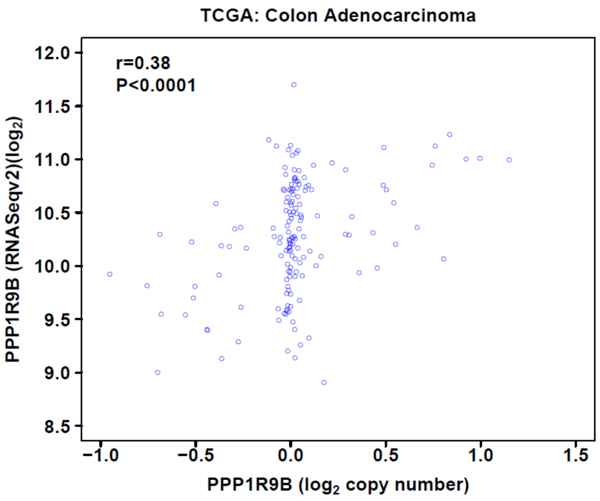

Supplementary Figure S4: Correlation between spinophilin expression and copy number changes in the TCGA dataset. A significant direct correlation (R = 0.38, p <0.001) between these two parameters could be identified.

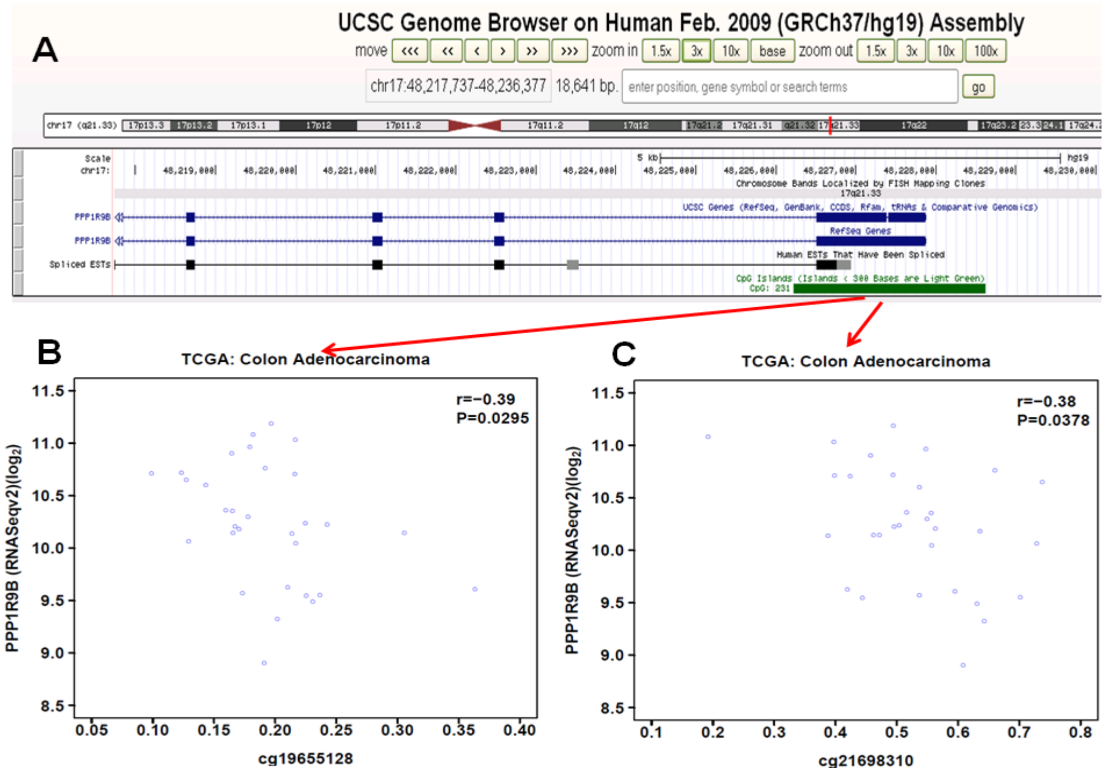

Supplementary Figure S5: CpG island in the spinophilin promoter region and DNA methylation. (A) Promoter region of spinophilin showing a closely related CpG island (Position: chr17:48226224-48228625, Band: 17q21.33, Genomic Size: 2402 (**B-C)** Two CpG sites in this region showed a significant negative correlation between spinophilin expression and beta-value of methylation. Note that PPP1R9B stands for spinophilin. X-axis: spinophilin expression, Y-axis: beta-value. The code used below the Figures (e.g. cg19655128) stands for the Illumina probes used.

Table S1: Association of clinico-pathological parameters and spinophilin expression in the study cohort (n=162)

Parameter	Low spinophilin expression	High spinophilin expression	p-value
**Localization**			
Right colon	27 (45.0)	41 (40.2)	
Transverse	5 (8.3)	8 (7.8)	0.460
Left colon	28 (46.7)	49 (48.0)	
n.a.	0 (0.0)	4 (3.9)	
**Gender**			
Female	27 (45.0)	53 (52.0)	0.392
male	33 (55.0)	49 (48.0)	
**Stage**			
n.a.	0 (0.0)	1 (1.0)	
Stage I	8 (13.3)	22 (21.6)	0.632
Stage II	23 (38.3)	38 (37.3)	
Stage III	18 (30.0)	25 (24.5)	
Stage IV	11 (18.3)	16 (15.7)	
**T-Stage**			
T1	1 (1.7)	3 (2.9)	
T2	12 (20.0)	22 (21.6)	0.919
T3	41 (68.3)	69 (67.6)	
T4	6 (10.0)	8 (7.8)	
**M-Stage**			
M0	48 (80.0)	85 (83.3)	0.838
M1	11 (18.3)	16 (15.7)	
n.a.	1 (1.7)	1 (1.0)	
**MSS**			
MSI-H	6 (10.0)	16 (15.7)	
MSI-L	10 (16.7)	22 (21.6)	0.369
MSS	44 (73.3)	64 (62.7)	

Legend
MSI: Microsatellite instable
MSS: Microsatellite stable
n.a. not available

Table S2: Association of clinico-pathological parameters and spinophilin expression in the study cohort (n=162)

Parameter	Low spinophilin expression	High spinophilin expression	p-value
**Localization**			
Right colon	27 (45.0)	41 (40.2)	0.460
Transverse	5 (8.3)	8 (7.8)	
Left colon	28 (46.7)	49 (48.0)	
n.a.	0 (0.0)	4 (3.9)	
**Age**			
<65	15 (25.0)	35 (34.3)	0.215
>65	45 (75.0)	67 (65.7)	
**Gender**	27 (45.0)	53 (52.0)	0.392
Female	33 (55.0)	49 (48.0)	
male			
**Stage**			
n.a.	0 (0.0)	1 (1.0)	
Stage I	8 (13.3)	22 (21.6)	0.632
Stage II	23 (38.3)	38 (37.3)	
Stage III	18 (30.0)	25 (24.5)	
Stage IV	11 (18.3)	16 (15.7)	
**T-Stage**			
T1	1 (1.7)	3 (2.9)	0.919
T2	12 (20.0)	22 (21.6)	
T3	41 (68.3)	69 (67.6)	
T4	6 (10.0)	8 (7.8)	
**M-Stage**			
M0	48 (80.0)	85 (83.3)	0.838
M1	11 (18.3)	16 (15.7)	
n.a.	1 (1.7)	1 (1.0)	
**MSS**			
MSI-H	6 (10.0)	16 (15.7)	0.369
MSI-L	10 (16.7)	22 (21.6)	
MSS	44 (73.3)	64 (62.7)	

Legend

MSI: Microsatellite instable

MSS: Microsatellite stable

n.a. not available
